# Estimation of Genetic Parameters of Early Growth Traits in Dumeng Sheep

**DOI:** 10.3390/ani14162298

**Published:** 2024-08-07

**Authors:** Ruijun Wang, Xinle Wang, Baodong Liu, Lifei Zhang, Jing Li, Dayong Chen, Yunhui Ma, Huijie He, Jie Liu, Yongbin Liu, Yanjun Zhang

**Affiliations:** 1 College of Animal Science, Inner Mongolia Agricultural University, Hohhot 010018, China; nmgwrj@126.com (R.W.); wangxinle163@163.com (X.W.); liubaodong302@163.com (B.L.); zhanglifei11220327@163.com (L.Z.); 18047916546@163.com (J.L.); 2 Inner Mongolia Sino Sheep Technology Co., Ltd., Ulanqab 011800, China; chendayong81@126.com (D.C.); m13847425547@163.com (Y.M.); 15904749394@163.com (H.H.); 15147461214@163.com (J.L.); 3 School of Life Sciences, Inner Mongolia University, Hohhot 010020, China; 4 Key Laboratory of Mutton Sheep Genetics and Breeding, Ministry of Agriculture, Hohhot 010018, China; 5 Key Laboratory of Goat and Sheep Genetics, Breeding and Reproduction, Inner Mongolia Autonomous Region, Hohhot 010018, China

**Keywords:** Dumeng sheep, genetic parameter, early growth traits, genetic correlation

## Abstract

**Simple Summary:**

Dumeng sheep is a novel, specialized meat sheep breed created through crossbreeding innovation, cross-fixation, and herd expansion, using Dorper sheep as the father and Mongolian sheep as the mother. Given the critical role of early growth and development stages in determining the meat production potential of livestock animals, estimating the genetic parameters of early growth traits is crucial for the genetic advancement of meat sheep populations. This study aimed to investigate the genetic and non-genetic factors that affect the nine early growth traits of Dumeng sheep, as well as to estimate the variance components and genetic parameters associated with these traits. It was found that the growth traits of Dumeng sheep could be improved indirectly by selecting for weaning weight.

**Abstract:**

This study aimed to estimate the genetic and non-genetic factors that affect the nine early growth traits of Dumeng sheep, as well as to estimate the variance components and genetic parameters associated with these traits. A dataset containing detailed information on 17,896 preweaning trait records of 4474 lambs was collected. In addition, 5015 postweaning trait records of 1003 lambs were documented. The effects of recipient dam age, sex, year, season, and herd on the early growth traits were assessed using the general linear model procedure of the statistical analysis system, revealing different levels of significance across different traits. To determine the most suitable model for estimating the genetic parameters, the likelihood ratio (LR) test was employed, fitting six animal models that either excluded or included maternal genetic and maternal permanent environmental effects within the average information restricted maximum likelihood (AIREML) framework using WOMBAT software (Version: 23/11/23). The model incorporating direct additive genetic effects, maternal genetic effects, and maternal permanent environment effects as random effects (model 6) provided the best fit for birth weight (BW) estimation. In contrast, the model combining direct additive genetic effects and maternal permanent environment effects as random effects (model 2) demonstrated a superior fit for estimating the genetic parameters of weaning weight (WW), average daily gain weight from birth to weaning (ADG1), and Kleiber ratio from birth to weaning (KR1). With regard to the genetic parameters of body weight at 6 months of age (6MW), average daily gain weight from weaning to 6 months (ADG2), average daily gain weight from birth to 6 months (ADG3), Kleiber ratio from weaning to 6 months (KR2), and Kleiber ratio from birth to 6 months (KR3), model 1, which incorporates only direct additive genetic effects, was identified as the optimal choice. With the optimal model, the heritability estimates ranged from 0.010 ± 0.033 for 6MW to 0.1837 ± 0.096 for KR3. The bivariate analysis method was employed to estimate the correlation between various traits using the most suitable model. The absolute values of genetic correlation coefficients among the traits spanned a range from 0.1460 to 0.9998, highlighting both weak and strong relationships among the studied traits. Specifically, the estimated genetic correlations between WW and ADG1, ADG3, KR1, and KR3 were 0.9859, 0.9953, 0.9911, and 0.9951, respectively, while the corresponding phenotypic correlations were 0.9752, 0.7836, 0.8262, and 0.5767. These findings identified that WW could serve as an effective selection criterion for enhancing early growth traits.

## 1. Introduction

Lamb is a nutritious food, rich in protein, amino acids, vitamins, and minerals. Compared to other meats, lamb is low in fat and cholesterol, making it a healthy choice [1]. With improved living standards upgraded, consumption structures, and increased health awareness in society, 40% of Chinese people have grown to prefer lamb meat because it is fresher and more tender than adult meat [2,3]. The demand for lamb has greatly contributed to the development of the meat sheep industry. China has 44 native sheep breeds, 32 cultivated (developed crossbred and artificial systematic selection) sheep breeds, and 13 introduced sheep breeds. Despite the presence of diverse and excellent local breeds, current lamb production is unable to meet consumer needs. To ensure sustainable and healthy growth in the meat sheep breeding industry, it is crucial to expedite the selection and breeding of high-quality breeding sheep. Significant progress has been made in breed improvement through the introduction and domestication of high-quality international meat sheep breeds, hybrid breeding to capitalize on hybrid vigor, and the development of highly productive meat sheep breeds [4]. The rate of breed improvement exceeds 60%, unlocking considerable potential for increased production and enhancing the quality of sheep meat products. The use of high-yield, adaptable breeds like Boer goats [5], Dorper sheep [6], and Suffolk sheep [7] in China has significantly increased lamb output and improved its quality.

Dumeng sheep is a recently developed, specialized meat sheep breed that has been produced through innovative crossbreeding innovation, cross-fixation, and herd expansion initiatives. Derived from crosses between Dorper rams with Mongolian ewes, this breed embodies rapid growth, high yield, and a lean meat ratio akin to Dorper sheep, coupled with the robust adaptability and superior meat qualities inherent in Mongolian sheep [6,8]. Its widespread appeal and dissemination across regions, including Inner Mongolia, Shandong, Ningxia, Gansu, and Xinjiang, attest to its tailored breeding objectives catering to the premium lamb market.

In light of the increasing demand for superior lamb, it is of paramount importance to elucidate the genetic basis of growth traits and to accurately assess genetic parameters, such as heritability and genetic correlations. Early growth traits include birth weight, weaning weight, and daily weight gain [9]. The selection of birth weight and weaning weight is crucial for the early selection of breeders and can significantly accelerate the progress of selection. These traits are affected by a complex of factors, including genetic and environmental factors. Genetic factors are the basis for determining the growth potential of livestock, which involves the genotype and genetic background of the livestock [10]. Livestock of different breeds and lines may differ significantly in growth rate, size, and adaptability. Therefore, by selecting livestock breeds with excellent genetic characteristics, the growth and development of their offspring can be effectively enhanced. Environmental influences on animal behavior include maternal factors, such as hormone levels and health status, as well as external environmental factors, such as season, birth type, birth year, dam age, and sex all significantly affecting growth traits in sheep [11,12]. Previous studies have shown that genetic variation exists for a range of growth traits among different sheep breeds, with heritability estimates ranging from low to high. For instance, published estimates of the heritability of WW include 0.21 in Mecheri sheep [13], 0.168 in Awassi sheep [14], and 0.20 in Sangsari sheep [15]. When devising genetic improvement schemes for lamb growth traits, comprehensive consideration of genetic and non-genetic influencers, prudent assessment of maternal effects, and careful avoidance of disregarding maternal additive genetic and permanent environmental impacts are vital to avoid biased variance estimates. This study aimed to estimate genetic parameters for traits before weaning and from weaning to six months and scrutinize genetic correlations among growth traits of each trait for integration into the genetic selection scheme.

## 2. Materials and Methods

### 2.1. Ethical Treatment

All animal experiments were conducted in compliance with the Guidelines for Experimental Animals established by the Ministry of Science and Technology (Beijing, China). This study was reviewed and approved by the Scientific Research and Academic Ethics Committee and the Biomedical Research Ethics Committee of Inner Mongolia Agricultural University ([2020] 056).

### 2.2. Animals and Data Collection

**Figure 1:**
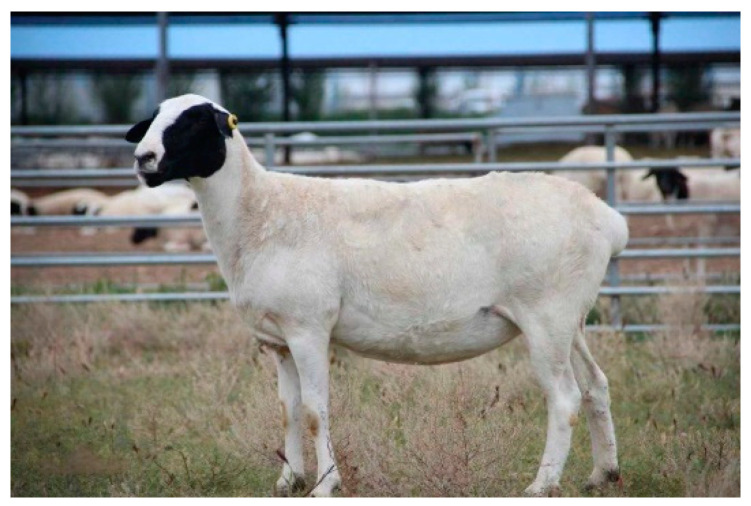
Dumeng sheep were used in this study.

The Dumeng sheep population is mostly raised in Ulanqab (Inner Mongolia, China). The central production area is located in the arid and semi-arid semi-desert steppe zone spanning 110°20′–113°00′ E and 41°10′−43°22′ N and belongs to the continental monsoon climate zone. The average annual precipitation is 313.8 mm, and 60–70% of the precipitation is concentrated in summer. The average climate of the hottest month is also generally below 20 °C. The present study utilized phenotypic data and pedigree information collected from 2019 to 2022 by the Inner Mongolia Sano Sheep Breeding Co., Ltd. (Ulanqab, China). Outliers (|*x*| > *x* ± 3*s.d*; *x* indicates the observational value of traits) and records from animals with missing or incorrect ear numbers, unclear sex, lack of key production performance data, or incomplete phenotypic information were omitted. Finally, 4474 lambs records from a detailed pedigree of 9 sires and 620 dams were used in this study (Figure 1). The study herd is distributed across 14 farms, with the number of individuals in each farm ranging from 82 to 1040 sheep. The sheep were grouped and housed in half-open-floor pens. The newborn lambs were weighed, plastic ear-tagged at birth, and weaned for about three months. The lambs were provided with ad libitum access to hay during the day and were allowed to suckle from their dams during the night until they reached 30 days of age. Two months after birth, the lambs were permitted to suckle their mothers twice a day, in the morning and evening, until they were weaned. Ninety-day weaned lambs are combined into one herd for uniform feeding management. The sheep were bred by artificial insemination and LOPU-IVF-ET at about 18 months of age. In the present study, the birth weight (BW), weaning weight (WW), average daily gain weight from birth to weaning (ADG1), preweaning Kleiber ratio (KR1), body weight at 6 months of age (6MW), average daily gain weight from weaning to 6 months (ADG2), average daily gain from birth to 6 months (ADG3), Kleiber ratio from weaning to 6 months (KR2), and Kleiber ratio from birth to 6 months (KR3) were analyzed. Since the lambs were weaned in the same batch, the weaning weights were corrected to 90 days of age. Similarly, due to the different age of measurement at 6 months of age, the weights were corrected to 180 days of age using the following equations [16,17,18]:

$$\begin{array}{c} ADG1=\frac{W2-W1}{D}\times 1000 \end{array}$$

$$\begin{array}{c} ADG2=\frac{W3-W2}{D}\times 1000 \end{array}$$

$$\begin{array}{c} ADG3=\frac{W3-W1}{D}\times 1000 \end{array}$$

$$\begin{array}{c} KR1=\frac{ADG1}{{WW}^{0.75}} \end{array}$$

WW=BW+ADG1×90

$$\begin{array}{c} 6MW=BW+ADG2\times 180 \end{array}$$

$$\begin{array}{c} KR2=\frac{ADG2}{{6MW}^{0.75}} \end{array}$$

$$KR3=\frac{ADG3}{{6MW}^{0.75}}$$

where W1 = BW, W2 = WW, W3 = 6MW, and D = number of days between weighting date and date of birth.

### 2.3. Statistical Analysis and Genetic Parameter Estimation

The studied environmental factors of each trait were determined by a general linear model (GLM) procedure using SAS software version 9.1 [19]. The studied environmental factors include the birth year of lambs (2019–2022), birth season (four seasons) of lambs, herd (fourteen doe flocks), sex (male or female), and age of recipient dam (2–5 years old). Duncan’s multiple range test was performed to compare the differences between various levels of the same significant factor. A statistical model of the GLM for the trait is described below:

(1) $$\begin{array}{c} y_{ijklmn}=\mu +Y_{i}+H_{j}+S_{k}+G_{l}+D_{m}+{YHM}_{ijk}+e_{ijklmn} \end{array}$$

where $y_{ijklmn}$ is the observation of *n*th kids that belonged to the *i*th year, the *j*th herd, the *k*th season, the *l*th gender, and the *m*th age of recipient dam. μ is the overall mean; ${YHM}_{ijk}$ is the interaction between the *i*th year, the *j*th herd, and the *k*th season; and $e_{ijklmn}$ is the random error.

The (co)variance components for each trait of six different animal models were estimated by the average information restricted maximum likelihood (AIREML) method, which was conducted using the WOMBAT program [20]. The models are as follows [21]:

(2) $$\begin{array}{c} y=Xb+Z_{1}a+e \end{array}$$

(3) $$\begin{array}{c} y=Xb+Z_{1}a+Z_{3}c+e \end{array}$$

(4) $$\begin{array}{c} y=Xb+Z_{1}a+Z_{2}m+e\;with\;COV\left(a,m\right)=0 \end{array}$$

(5) $$\begin{array}{c} y=Xb+Z_{1}a+Z_{2}m+e\;with\;COV\left(a,m\right)=A\sigma_{am} \end{array}$$

(6) $$\begin{array}{c} y=Xb+Z_{1}a+Z_{2}m+Z_{3}c+e\;with\;COV\left(a,m\right)=0 \end{array}$$

(7) $$\begin{array}{c} y=Xb+Z_{1}a+Z_{2}m+Z_{3}c+e\;with\;COV\left(a,m\right)=A\sigma_{am} \end{array}$$

where y is a vector of observed traits; b, a, m, c are vectors of fixed effects, direct additive genetic effects, maternal additive genetic effects, maternal permanent environmental effects, and vector, respectively. e is residual effects; X, $Z_{1}$, $Z_{2}$, and $Z_{3}$ are structure matrices, respectively, relating b, a, m, and c to y. The (co)variance structure of the random effects was as follows [17]:

(8) $$\begin{array}{c} Var\left(a\right)=A\sigma_{a}^{2},\;Var\left(m\right)=A\sigma_{m}^{2},\;Var\left(c\right)=I_{d}\sigma_{c}^{2},\;Var\left(e\right)=I_{n}\sigma_{e}^{2}\;and\;COV\left(a,m\right)=A\sigma_{am} \end{array}$$

Direct additive and maternal genetic effects were assumed to be distributed normally with a mean of 0 and variances of $A\sigma_{a}^{2}$ and $A\sigma_{m}^{2}$ covariance structure, respectively. Where $\sigma_{a}^{2}$, $\sigma_{m}^{2}$, $\sigma_{c}^{2}$, $\sigma_{e}^{2}$, and $\sigma_{am}$ are the direct additive genetic variance, maternal additive genetic variance, maternal permanent environmental variance, residual variance, and the covariance between the direct additive genetic and maternal additive genetic effect, respectively. A represents the additive genetic correlation matrix, and $I_{d}$ and $I_{n}$ are identity matrices of order equal to the number of dams and the number of lambs, respectively. All components with the phenotypic variance ($\sigma_{p}^{2}$) being the sum of $\sigma_{a}^{2}$, $\sigma_{m}^{2}$, $\sigma_{c}^{2}$, and $\sigma_{e}^{2}$ were derived at convergence [22].

The direct heritability:

(9) $$\begin{array}{c} h_{d}^{2}=\frac{\sigma_{a}^{2}}{\sigma_{p}^{2}} \end{array}$$

The maternal heritability:

(10) $$\begin{array}{c} h_{m}^{2}=\frac{\sigma_{m}^{2}}{\sigma_{p}^{2}} \end{array}$$

The maternal permanent environmental heritability:

(11) $$\begin{array}{c} c^{2}=\frac{\sigma_{c}^{2}}{\sigma_{p}^{2}} \end{array}$$

The genetic correlation between the direct and maternal effects:

(12) $$\begin{array}{c} r_{am}=\frac{\sigma_{am}}{\sqrt{\sigma_{a}^{2}{*\sigma }_{m}^{2}}} \end{array}$$

The direct–maternal correlation ($r_{am}$) was computed as the ratio of the estimates of the direct maternal covariance ($\sigma_{am}$) to the product of the square roots of the estimates of $\sigma_{a}^{2}$ and $\sigma_{m}^{2}$. The test of the accuracy of the estimation of the variance components of the different models can be evaluated using the *AIC* information criterion as an evaluation criterion. The AIC is calculated as follows [23]:

(13) $$\begin{array}{c} AIC=2k-2logL \end{array}$$

The likelihood ratio test (LR) was performed to obtain the appropriate model as given below [24]:

(14) $$\begin{array}{c} LR=-2\log\frac{L_{1}}{L_{2}}=-2\left[\log\left(L_{1}\right)\right]-\left[\log\left(L_{2}\right)\right] \end{array}$$

where $L_{1}$ and $L_{2}$ represent the maximum likelihood function values of model 1 and model 2, respectively. Model 1 is a sub-model of model 2. The LR follows the chi-square distribution, with the degree of freedom equal to the number of parameters considered in model 2 minus the number of parameters in model 1. Furthermore, the genetic and phenotypic correlations between the traits were obtained using bivariate animal models based on the most appropriate model for each trait. The bivariate model is as follows [3]:

(15) $$\begin{array}{c} \left[\begin{array}{c} y_{1}\\ y_{2} \end{array}\right]=\left[\begin{array}{cc} X_{1} & 0\\ 0 & X_{2} \end{array}\right]\left[\begin{array}{c} b_{1}\\ b_{2} \end{array}\right]+\left[\begin{array}{cc} Z_{1} & 0\\ 0 & Z_{2} \end{array}\right]\left[\begin{array}{c} a_{1}\\ a_{2} \end{array}\right]+\left[\begin{array}{c} e_{1}\\ e_{2} \end{array}\right] \end{array}$$

where and $y_{1}$ and $y_{2}$ is the vector of phenotypic values for traits 1 and 2, $b_{1}$ and $b_{2}$ is the vector of fixed effects for $a_{1}$ and$\;a_{2}$, $a_{1}$ and$\;a_{2}$ is the vector of random animal genetic effects, $e_{1}$ and$\;e_{2}\;$is the vector of random residuals, $X_{1}$ and $X_{2}$ are the design matrices for fixed effects, and $Z_{1}\;$and $Z_{2}$ are the design matrices for the traits of interest with random animal genetic effects. The genetic correlation and phenotypic correlation between the two traits were estimated according to the method of Alam et al. [25]:

(16) $$\begin{array}{c} r_{A}=\frac{Cov\left(a_{1},a_{2}\right)}{\sqrt{\sigma_{a_{1}}^{2}\sigma_{a_{2}}^{2}}} \end{array}$$

(17) $$\begin{array}{c} r_{P}=\frac{Cov\left(p_{1},p_{2}\right)}{\sqrt{\sigma_{p_{1}}^{2}\sigma_{p_{2}}^{2}}} \end{array}$$

where $Cov\left(a_{1},a_{2}\right)$ and $Cov\left(p_{1},p_{2}\right)$ are the genetic and phenotypic covariance between two traits, respectively, and the $\sigma_{a_{1}}^{2}$, $\sigma_{a_{2}}^{2}$, $\sigma_{p_{1}}^{2}$, and $\sigma_{p_{2}}^{2}$ parameters are genetic and phenotypic variance estimates of trait 1 and 2, respectively.

## 3. Results

### 3.1. Descriptive Statistics

Table 1 shows the details of the pedigree of the Dumeng sheep. A total of 4474 newborn lambs from 9 rams and 620 ewes were recorded for preweaning traits, and 1003 lambs from 8 rams and 297 ewes were recorded for postweaning traits. The average number of records per ewe ranged from 3.3 (postweaning traits) to 7.2 (preweaning traits), while rams had values ranging from 125 to 497. The pedigree constitution check revealed enough information to estimate genetic components. From Table 1, the coefficient of variation of BW, WW, ADG1, KR1, 6MW, ADG2, KR2, ADG3, and KR3 reached 27%, 18%, 21%, 8%, 16%, 34%, 18%, 28%, and 6%, respectively. These figures underscore the extent of variability within the herd, which is pivotal for genetic evaluation and improvement programs.

### 3.2. Environmental Effects of Earth Growth Traits

Table 2 shows the least square mean of each trait and its standard error. The overall least square means ± SE for BW, WW, ADG1, and KR1 of Dumeng sheep were 4.20 ± 0.02, 31.39 ± 0.08, 302.08 ± 0.94, and 22.63 ± 0.03, respectively. The results showed that the age of recipient dam, sex, birth year, birth season, and herd have a very significant impact on four traits. The interaction of birth year, birth season, and herd had a significant impact on four traits. Therefore, birth year, birth season, and herd are combined to form a new variable By*Bs*Herd in the model for estimating these four traits, which is composed of different levels of birth year, birth season, and herd. Similarly, the overall least square means ± SE for 6MW, ADG2, KR2, ADG3, and KR3 for Dumeng sheep were 53.93 ± 0.28, 232.20 ± 2.47, 11.58 ± 0.10, 274.55 ± 1.54, and 13.74 ± 0.03, respectively (Table 3). The results showed that age of recipient dam, sex, birth year, and birth season have a very significant impact on five traits.

### 3.3. Variance Components and Genetic Parameter Estimates

The variance components and genetic parameters of BW, WW, ADG1, KR1, 6MW, ADG2, KR2, ADG3, and KR3 estimated by the six models are listed in Table 4. The variance components estimated by different models for the same trait were quite different. With the inclusion of the maternal genetic effect, the maternal environmental effect, or the direct additive and maternal additive genetic covariance in other models, the direct additive genetic variance and heritability estimates changed. The likelihood ratio test results of the different models are listed in Table 5. There were significant differences among the models regarding BW traits. It showed that the most suitable model of BW is model 6, which should include direct additive genetic effects, maternal genetic and environmental effects, and direct–maternal additive genetic correlation. A comparison of the different models for the WW, ADG1, and KR1 traits showed that increasing the parent permanent environment effect in the model significantly improved the model’s goodness of fit, with model 2 outperforming the other models. For the comparison of different models for the 6MW, ADG2, ADG3, KR2, and KR3 traits, the results showed that the likelihood ratio test was not significant, and no significant model improvement was seen when maternal permanent environment effects, direct additive genetic effects, maternal genetic effects, and direct maternal covariates were added to the model comparison. Therefore, model 1 was selected as the 6MW, ADG2, ADG3, KR2, and KR3 traits as the best animal model. The results of the likelihood ratio test between different models were consistent with the results of the optimal model derived from the *AIC* results.

As presented in Table 4, the heritabilities of BW, WW, ADG1, KR1, 6MW, ADG2, KR2, ADG3, and KR3 were estimated to be 0.0352, 0.0446, 0.0564, 0.0724, 0.010, 0.123, 0.058, 0.1826, and 0.1837, respectively, using optimal models for each trait, which belonged to low-to-medium heritability. Among the optimal models for each trait, only BW had a maternal heritability effect of 0.1375, which is a medium heritability. In addition to maternal heritability, the maternal permanent environmental effects of BW, WW, ADG1, and KR1 belong to medium–high heritability with 0.4907, 0.2153, 0.1915, and 0.1474, respectively. The correlation of the direct genetic effect of BW with the maternal genetic effect was −0.9838, which was a strong negative correlation.

### 3.4. Heritability, Genetic and Phenotypic Correlation among Traits

**Figure 2:**
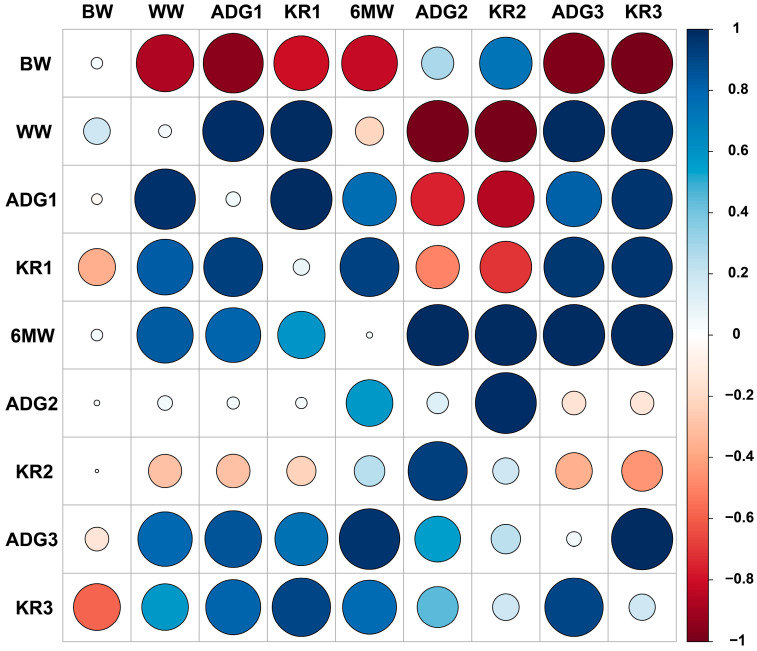
Heritability, genetic, and phenotypic correlation between early growth traits in Dumeng sheep.

Large sample sizes are often required to accurately estimate genetic correlations in animal breeding, since they are often subjected to large sampling errors [26]. Several bivariate analyses were performed to estimate the heritability, genetic, and phenotypic correlations between the growth traits of Dumeng sheep (Figure 2). The results of the analyses are represented in Table 6. The phenotypic and genetic correlation estimates between most of the growth traits of Dumeng sheep were strongly positive. ADG1 had a positive genetic correlation with KR1 (0.9964), 6MW (0.7623), ADG3 (0.8119), and KR3 (0.9645). 6MW also had a positive genetic correlation with ADG2 (0.9978), KR2 (0.9929), ADG3 (0.9998), and KR3 (0.9998). Unexpectedly, there were antagonistic phenotypic and genetic relationships between BW and ADG1, KR1, ADG3, and KR3, suggesting that selection for BW may reduce these four traits. On the other hand, high and positive genetic correlations were observed among WW and other growth traits (ADG1, KR1, 6MW, ADG3, and KR3 traits), so indirect selection for ADG and KR could be achieved by selecting WW, which gained its own faster genetic progression.

## 4. Discussion

The Dumeng sheep is a new breed bred from crossbreeding Dupor and Mongolian sheep, characterized by a fast growth rate, high meat production performance, and adaptation to desert and semi-desert grassland environments [6,8]. Early growth traits mainly include birth weight, weaning weight, and daily weight gain. Kleiber’s ratio responds to the rate and efficiency of an animal’s growth and development during a given growth phase, providing an important indicator for selection decisions. As growth traits are typically difficult and expensive to measure, no studies have reported genetic parameters for growth traits in Dumeng sheep to our knowledge [3]. Estimating the genetic parameters of early growth traits in Dumeng sheep is of great significance for understanding its genetic basis, formulating effective selection and breeding strategies, and improving the production performance of the whole breed in this study. Especially in the current context of pursuing efficient and environmentally friendly animal husbandry, precision breeding is particularly important.

### 4.1. Environmental Effects

With the continuous development of biotechnology, there is a greater need for high-quality trait selection in biological breeding. However, the phenotypic characteristics of traits such as growth and development are influenced not only by parental genes but also by environmental (non-genetic) factors such as year, season, sex, herd, etc. [27]. The coefficient of variation of each growth trait was between 6 and 34% in this study, indicating that each character had a certain degree of phenotypic variation. The results revealed that birth season have significant effects on BW, WW, ADG1, and KR1 (*p* < 0.01) in the present study and were in agreement with the previous reports on Norduz lambs [12] and Iranian Zandi sheep [28]. The effect of herd has been reported to be significant in breeds like Merino lambs [29] and Kilis goats [30] (*p* < 0.01). In addition, Dhakad et al. [31] and Mohammadi et al. [28] showed that season also significantly affected the mean postweaning weight. Due to the different lambing seasons, climate change varies greatly, which will have a certain effect on the growth and development of the lambs; generally speaking, spring lambing is better than the lambs born in the fall and winter seasons [32]. Male lambs were heavier than female lambs in all BW. One possible reason may be due to hormonal and physiological differences in the two sexes. The significant effects of sex may be attributed to several reasons such as the difference in the endocrine systems of female and male kids [22]. The interaction of birth year, birth season, and herd had a significant impact on each trait.

### 4.2. Model Comparisons

The likelihood ratio test showed that there were significant differences between the models of BW; the most suitable model of BW is model 6 and should include the effects of direct additive genetic effects, maternal permanent environmental effects, maternal additive genetic effects, and the direct–maternal interaction effect. The effect of maternal genetics was the greatest at birth, but the effect of maternal genetics would gradually decrease as the age of the day increased. Aguirre [33] et al. in the estimation of variance components in Santa Ines sheep population concluded that the weight trait increases with age, with a significant effect of maternal effect in the preweaning stage and a decrease in the effect after weaning [34]. Comparison of the different models for the WW, ADG1, and KR1 traits showed that these traits, with model 2 outperforming the other models, increasing the maternal permanent environment effect in the model significantly improved the model’s goodness of fit. These three traits are affected not only by the genetic potential of the individual’s growth and development but also by the permanent environmental effects of the ewes. Because the lambs in this study were raised using the reproductive technique of in vitro fertilization with embryo transfer, environmental effects on early growth traits may be due to the different environments of the embryos during pregnancy or lactation in the recipient ewes. The results of Kushwaha [35] and others showed that the estimates of direct heritability for all traits were significantly overstated without considering maternal effects, and that more accurate estimates could only be obtained after different model comparisons when both maternal additive and maternal permanent environmental effects were taken into account within the model. Similar to the results of the present study, direct selection for preweaning traits leads to indirect selection for maternal effects, which are particularly important for early growth traits in sheep as the selection an effect during pregnancy and lactation. For different model comparisons of 6MW, ADG2, ADG3, KR2, and KR3 traits, model 1 served as the best animal model for these traits. After adding maternal permanent environment effects, direct additive genetic effects, maternal genetic effects, and direct–maternal covariates to the model comparisons, the likelihood ratio test was not significant and the models did not improve significantly.

### 4.3. Genetic Parameters Estimate

These findings align with reports by Ahmad [36] et al. and Magotra [37] et al., who found low heritability for newborn weight (0.130 and 0.006) in Corriedale sheep and Beetal goats, respectively, while observing moderate heritability for WW and 6MW. The consistently low heritability of lamb birth weight suggests that non-genetic factors, predominantly environmental influences, play a significant role in determining variations in this trait. In contrast, Tesema et al. [38] reported higher heritability estimates for Boer goats: 0.38 for BW, 0.12 for WW, and 0.05 for 6MW, with daily weight gains showing heritability between 0.08 and 0.09. The heritability of KR at different growth stages ranged from low to moderate heritability (0.09 to 0.18). The medium heritability for BW was different from the results of the present study, whereas the low heritability for all other growth traits was consistent with the present study. Differences in heritability might stem from environmental disparities given Boer goats inhabit plateau regions. Singh et al. [39] estimated the growth traits of Barbari goats based on LR and the direct heritability of the best model to estimate ADG and KR values at different stages of growth were of moderate heritability, and the above estimates were higher than those of the present study, potentially influenced by the differential impact of environmental factors or a greater genetic diversity affecting heritability estimations. Sharif et al. [11] reported direct heritability estimates of 0.15, 0.20, and 0.20 for birth weight, 120-day weight, and 180-day weight in Lohi Sheep, respectively, reflecting low to moderate heritability, diverging from our findings, possibly due to distinct environmental impacts on different breeds. Besufkad et al. [40] genetically evaluated traits related to growth rate and efficiency in a population of Dupor crossbred sheep and the estimates of direct heritability for birth to weaning ADG and weaning to 6 months of ADG, and the corresponding KRs were 0.45 ± 0.15, 0.04 ± 0.06, 0.30 ± 0.08, 0.13 ± 0.11, respectively, with ADG and KR from birth to weaning day being of medium heritability, which was different from the results of the present study, and from weaning to 6 months of ADG and KR being of low heritability, which was consistent with the results of the present study. The reason for the difference may be related to the size of the group; the larger the group, the more accurate the heritability estimation will be, and if the group is small, it will be limited. The group of Doper sheep was only 1350 less than the number of individuals in the present study. The error is larger when the group size is small and is easily affected by the proportion of individual variance and residuals; the larger the sample, the more the estimation tends to stabilize, and the smaller the effect.

The maternal heritability values of BW estimated in this study were 0.1375, which were lower than those reported by Mohammadi et al. [28] for Raeini cashmere goats (0.17 for BW), Zhang et al. [41] for Boer goats (0.26 for BW), and Buxadera et al. [42] for Creole goats (0.24 for BW). The lower maternal heritability and maternal permanent environmental effects (0.4907 for BW) may be because the effects considered for each trait in this study were more than those in other studies.

### 4.4. Correlation Estimates

Genetic and phenotypic correlations among traits are pivotal in refining breeding strategies, enabling selection based on high coefficients of both genetic and phenotypic correlations. Genetic correlations, reflecting interactions between genetic elements, are genuinely heritable, whereas phenotypic correlations, typically influenced by environmental factors such as feeding regimens and nutrition, do not convey authentic heritability to offspring [43]. Variations in traits within a breed can be attributed to several factors: Firstly, quantitative traits are largely influenced by micro-effect polygenes susceptible to environmental impacts. Different breeds, subjected to varying environmental conditions and selective pressures, exhibit diverse gene expressions despite shared genetics, leading to trait-specific genetic effect disparities. Secondly, genetic correlations arise from pleiotropy and genetic linkage, implying that the number of shared genes, the specific genes governing different traits, and the strength of gene linkages significantly contribute to dissimilarities in trait genetic correlations. Lastly, the statistical models, dataset sizes, and estimation methodologies employed in analyses contribute to variations in parameter estimations for identical traits across breeds or different traits within the same breed.

In this study, examining early growth traits in Dumeng sheep, we observed strong positive correlations between WW and ADG1, ADG3, and their respective KR1, KR3, suggesting indirect selection for ADG and KR through WW. Magotra et al. [37] posited that negative genetic correlations between birth weight and postweaning growth traits may result from reduced maternal genetic effects and enhanced additive genetic variance. Phenotypic correlations among all studied traits were positive, likely due to a shared environment. Ahmad et al. [36] emphasized the high correlation of WW and 6MW with other traits in Corriedale sheep, advocating for prioritizing these traits in selection schemes to achieve moderate genetic progress. Practically, WW is favored due to its earlier manifestation in production, where early trait expression responds better to selection, aligning with Shokrollahi et al.’s [44] findings in Arabi sheep. Ofori et al. [45] noted a wide range of phenotypic correlations (0.04 to 0.95) and moderate to high genetic correlations (0.30 to 0.96) among West African dwarf goats’ traits, echoing our findings, particularly regarding the high genetic correlation between weaning weight and 6-month body weight. Singh et al. [39] found significant genetic correlations between ADG and KR in Barbari goats, supporting the use of KR as an indicator of improved feed efficiency. High genetic correlations among ADG, weaning weight, and KR, as seen in our study and echoed by Dige et al. [46] in Jamunapari goats, imply that selection of one trait could yield substantial advancements in related traits. Phenotypic correlations revealed beneficial associations among preweaning traits, implying that selecting for weaning weight could boost ADG and feed efficiency in Dumeng sheep.

## 5. Conclusions

The model 6 provided the best fit for BW estimation. In contrast, the model 2 demonstrated a superior fit for estimating genetic parameters of WW, ADG1, and KR1. With regard to the genetic parameters of 6MW, ADG2, ADG3, KR2, and KR3, model 1 was identified as the optimal choice. With the optimal model, the heritability estimates ranged from 0.010 ± 0.033 for 6MW to 0.1837 ± 0.096 for KR3. The absolute values of genetic correlation coefficients among the traits spanned a range from 0.1460 to 0.9998. Specifically, the estimated genetic correlations between WW with ADG1, ADG3, KR1, and KR3 were 0.9859, 0.9953, 0.9911, and 0.9951, respectively. While corresponding phenotypic correlations were 0.9752, 0.7836, 0.8262, and 0.5767. The low direct heritability estimates for the traits under investigation indicate inherent difficulties in genetic enhancement via direct selection. Nevertheless, the moderate to strong genetic correlations observed for ADG1, ADG3, KR1, and KR3 with weaning weight suggest that weaning weight may be a viable selection criterion for improving early growth traits.

## Figures and Tables

**Table 1:** Description of a data structure for early growth traits of Dumeng sheep.

Item	BW, kg	WW, kg	ADG1, g	KR1	6MW, kg	ADG2, g	ADG3, g	KR2	KR3
No. of records	4474	4474	4474	4474	1003	1003	1003	1003	1003
No. of sires	9	9	9	9	8	8	8	8	8
No. of dams	620	620	620	620	297	297	297	297	297
Average litter size of sire	497	497	497	497	125	125	125	125	125
Average litter size of dam	7.2	7.2	7.2	7.2	3.3	3.3	3.3	3.3	3.3
Mean	4.20	31.39	302.08	22.63	53.93	232.20	274.55	11.58	13.74
SD	1.12	5.57	63.16	1.90	8.73	78.31	48.92	3.22	0.87
CV (%)	27	18	21	8	16	34	18	28	6

BW: birth weight; WW: weaning weight; ADG1, average daily gain weight from birth to weaning; KR1: Kleiber ratio from birth to weaning (ADG1/WW^0.75^); 6MW: body weight at 6 months of age; ADG2: average daily gain weight from weaning to 6 months; ADG3: average daily gain weight from birth to 6 months; KR2: Kleiber ratio from weaning to 6 months; KR3: Kleiber ratio from birth to 6 months; Mean: average value; SD: standard deviation; CV: coefficient of variation.

**Table 2:** Least square means (±SE) for preweaning traits of Dumeng sheep.

Factors	*n*	BW, kg	WW, kg	ADG1, g	KR1
Overall	4474	4.20 ± 0.02	31.39 ± 0.08	302.08 ± 0.94	22.63 ± 0.03
Dam age		<0.001 ***	<0.001 ***	<0.001 ***	<0.001 ***
2	1032	4.37 ± 0.03 ^a^	28.94 ± 0.13 ^d^	272.64 ± 1.38 ^d^	21.78 ± 0.05 ^d^
3	977	4.17 ± 0.02 ^b^	30.85 ± 0.16 ^c^	296.50 ± 1.72 ^c^	22.54 ± 0.05 ^c^
4	2277	4.19 ± 0.03 ^b^	32.39 ± 0.12 ^b^	313.26 ± 1.40 ^b^	22.91 ± 0.04 ^b^
5	188	3.41 ± 0.07 ^c^	35.57 ± 0.48 ^a^	357.36 ± 5.45 ^a^	24.37 ± 0.14 ^a^
Sex		<0.001 ***	<0.001 ***	<0.001 ***	<0.001 ***
Male	2310	4.27 ± 0.02 ^a^	33.12 ± 0.12 ^a^	320.57 ± 1.31 ^a^	23.09 ± 0.04 ^a^
Female	2164	4.12 ± 0.02 ^b^	29.55 ± 0.11 ^b^	282.36 ± 1.23 ^b^	22.15 ± 0.04 ^b^
Birth year		<0.001 ***	<0.001 ***	<0.001 ***	<0.001 ***
2019	203	4.72 ± 0.07 ^a^	29.21 ± 0.26 ^c^	272.08 ± 2.84 ^d^	21.59 ± 0.09 ^d^
2020	1451	4.23 ± 0.02 ^c^	29.55 ± 0.12 ^c^	281.12 ± 1.29 ^c^	22.09 ± 0.04 ^c^
2021	2201	4.33 ± 0.03 ^b^	31.46 ± 0.11 ^b^	301.52 ± 1.27 ^b^	22.56 ± 0.04 ^b^
2022	619	3.48 ± 0.03 ^d^	36.16 ± 0.25 ^a^	363.08 ± 2.83 ^a^	24.48 ± 0.07 ^a^
Birth season		<0.001 ***	<0.001 ***	<0.001 ***	<0.001 ***
Spring	918	4.37 ± 0.03 ^a^	30.93 ± 0.17 ^b^	295.14 ± 1.84 ^b^	22.38 ± 0.05 ^b^
Summer	686	4.34 ± 0.03 ^a^	29.62 ± 0.16 ^d^	280.91 ± 1.75 ^d^	22.03 ± 0.06 ^c^
Autumn	847	4.05 ± 0.02 ^c^	30.13 ± 0.17 ^c^	289.83 ± 1.88 ^c^	22.42 ± 0.05 ^b^
Winter	2023	4.13 ± 0.03 ^b^	32.73 ± 0.13 ^a^	317.54 ± 1.57 ^a^	23.04 ± 0.05 ^a^
Herd		<0.001 ***	<0.001 ***	<0.001 ***	<0.001 ***
1	108	3.99 ± 0.10 ^gh^	36.68 ± 0.44 ^b^	363.24 ± 5.06 ^b^	24.29 ± 0.13 ^b^
2	108	4.23 ± 0.09 ^ef^	35.59 ± 0.39 ^c^	348.42 ± 4.36 ^c^	23.86 ± 0.12 ^c^
3	804	4.36 ± 0.03 ^de^	29.98 ± 0.12 ^gh^	284.64 ± 1.29 ^f^	22.17 ± 0.04 ^f^
4	188	7.35 ± 0.06 ^a^	31.09 ± 0.31 ^ef^	263.87 ± 3.28 ^gh^	19.94 ± 0.11 ^i^
5	272	4.15 ± 0.06 ^fg^	29.64 ± 0.25 ^gh^	283.29 ± 2.82 ^f^	22.21 ± 0.09 ^f^
6	82	5.13 ± 0.15 ^b^	29.32 ± 0.49 ^h^	268.74 ± 4.94 ^g^	21.26 ± 0.16 ^h^
7	253	4.02 ± 0.06 ^gh^	33.87 ± 0.33 ^c^	331.68 ± 3.67 ^d^	23.52 ± 0.10 ^d^
8	176	4.30 ± 0.08 ^def^	31.51 ± 0.33 ^e^	302.42 ± 3.89 ^e^	22.63 ± 0.13 ^e^
9	408	3.65 ± 0.03 ^i^	26.70 ± 0.19 ^i^	255.12 ± 2.04 ^h^	21.67 ± 0.08 ^g^
10	250	4.44 ± 0.05 ^d^	30.23 ± 0.24 ^gh^	286.49 ± 2.66 ^f^	22.16 ± 0.08 ^f^
11	193	4.67 ± 0.06 ^c^	29.91 ± 0.33 ^gh^	280.39 ± 3.68 ^f^	21.81 ± 0.12 ^g^
12	322	3.90 ± 0.07 ^h^	36.91 ± 0.35 ^b^	366.83 ± 3.85 ^b^	24.36 ± 0.10 ^b^
13	1040	3.90 ± 0.68 ^h^	30.50 ± 0.16 ^fg^	295.54 ± 1.83 ^e^	22.62 ± 0.06 ^e^
14	270	3.18 ± 0.35 ^j^	38.07 ± 0.34 ^a^	387.66 ± 3.78 ^a^	25.21 ± 0.08 ^a^
By*Bs*Herd		<0.001 ***	<0.001 ***	<0.001 ***	<0.001 ***

Note: Dam age: age of recipient dam; By*Bs*Herd: a combination of the year, season, and herd at birth. The means with different letters in each sub-class within a column differ significantly from another. *** *p* < 0.001.

**Table 3:** Least square means (±SE) for postweaning traits of Dumeng sheep.

Factors	*n*	6MW, kg	ADG2, g	KR2	ADG3, g	KR3
Overall	1003	53.93 ± 0.28	232.20 ± 2.47	11.58 ± 0.10	274.55 ± 1.54	13.74 ± 0.03
Dam age		<0.001 ***	<0.001 ***	<0.001 ***	<0.001 ***	<0.001 ***
2	284	48.79 ± 0.42 ^b^	203.17 ± 3.85 ^b^	10.91 ± 0.17 ^b^	246.09 ± 2.32 ^b^	13.30 ± 0.05 ^b^
3	80	56.44 ± 0.79 ^a^	214.66 ± 8.17 ^b^	10.40 ± 0.37 ^b^	288.92 ± 4.43 ^a^	13.99 ± 0.07 ^a^
4	623	55.90 ± 0.35 ^a^	246.80 ± 3.24 ^a^	12.00 ± 0.13 ^a^	285.34 ± 1.97 ^a^	13.90 ± 0.03 ^a^
5	16	56.00 ± 1.52 ^a^	266.28 ± 13.65 ^a^	12.99 ± 0.58 ^a^	287.58 ± 9.71 ^a^	14.01 ± 0.21 ^a^
Sex		<0.001 ***	<0.001 ***	0.0427 *	<0.001***	<0.001 ***
Male	584	57.95 ± 0.32 ^a^	249.20 ± 3.51 ^a^	11.78 ± 0.15 ^a^	296.46 ± 1.81 ^a^	14.07 ± 0.03 ^a^
Female	419	48.33 ± 0.33 ^b^	208.50 ± 2.96 ^b^	11.29 ± 0.13 ^b^	244.01 ± 1.88 ^b^	13.28 ± 0.04 ^b^
Birth year		<0.001 ***	<0.001 ***	<0.001 ***	<0.001***	<0.001 ***
2019	66	50.52 ± 0.83 ^b^	226.19 ± 9.37 ^b^	11.80 ± 0.37 ^a^	254.22 ± 37.21 ^b^	13.38 ± 0.08 ^b^
2020	280	50.27 ± 0.48 ^b^	204.96 ± 3.73 ^c^	10.79 ± 0.16 ^b^	254.86 ± 44.28 ^b^	13.46 ± 0.05 ^b^
2021	657	55.84 ± 0.34 ^a^	244.41 ± 3.18 ^a^	11.89 ± 0.13 ^a^	284.98 ± 48.66 ^a^	13.90 ± 0.03 ^a^
Birth season		<0.001 ***	<0.001 ***	<0.001 ***	<0.001***	<0.001 ***
Spring	232	54.61 ± 0.49 ^b^	169.62 ± 5.31 ^c^	8.43 ± 0.25 ^c^	276.56 ± 39.29 ^b^	13.74 ± 0.04 ^b^
Summer	141	54.18 ± 0.73 ^b^	231.30 ± 6.85 ^b^	11.51 ± 0.28 ^b^	275.96 ± 34.70 ^b^	13.77 ± 0.07 ^a^
Autumn	66	56.38 ± 0.66 ^a^	271.12 ± 4.96 ^a^	13.03 ± 0.17 ^a^	290.65 ± 37.21 ^a^	14.05 ± 0.06 ^b^
Winter	564	52.25 ± 0.37 ^c^	232.16 ± 3.10 ^b^	11.86 ± 0.13 ^b^	264.38 ± 50.75 ^c^	13.56 ± 0.04 ^a^

Note: The means with different letters in each sub-class within a column differ significantly from another. * *p* < 0.05, and *** *p* < 0.001.

**Table 4:** Estimates of the (co)variance components and the genetic parameters studied for the traits.

Traits	Models	$\sigma_{a}^{2}$	$\sigma_{m}^{2}$	$\sigma_{am}$	$\sigma_{c}^{2}$	$\sigma_{e}^{2}$	$\sigma_{p}^{2}$	$h_{d}^{2}\pm S.E.$	$h_{m}^{2}\pm S.E.$	$c^{2}\pm S.E.$	$r_{am}\pm S.E.$	−2log⁡L	*AIC*
BW	model1	0.0412				0.8022	0.8433	0.0488 ± 0.027				3798.326	3802.326
	model2	0.0525			0.4492	0.3722	0.8741	0.0601 ± 0.027		0.514 ± 0.027		3681.85	3687.854
	model3	0.0067	0.0562			0.7825	0.8455	0.008 ± 0.010	0.0665 ± 0.014			3782.862	3788.862
	model4	0.0323	0.1375	−0.0656		0.7575	0.8617	0.0375 ± 0.049	0.1596 ± 0.048		−0.984 ± 0.475	3777.286	3785.286
	model5	0.0049	0.0539		0.4401	0.3742	0.8722	0.0056 ± 0.009	0.0608 ± 0.013	0.5046 ± 0.027		3664.378	3672.378
	model6	0.0311	0.1217	−0.0606	0.4342	0.3584	0.5294	0.0352 ± 0.049	0.1375 ± 0.046	0.4907 ± 0.027	−0.9838 ± 0.511	3659.218	3669.218
WW	model1	0.9704				16.85	17.821	0.0545 ± 0.024				17,333.78	17,337.78
	model2	0.7913			3.8197	13.129	17.74	0.0446 ± 0.023		0.2153 ± 0.031		17,282.722	17,288.722
	model3	0.9745	0.001			16.849	17.824	0.0547 ± 0.043	0.0001 ± 0.013			17,333.784	17,339.784
	model4	1.0311	0.0014	−0.0195		16.823	17.836	0.0578 ± 0.057	0.0001 ± 0.058		−0.520 ± failed	17,333.776	17,341.776
	model5	0.8021	0.001		3.8203	13.125	17.748	0.0452 ± 0.037	0.0001 ± 0.012	0.2153 ± 0.031		17,282.73	17,290.73
	model6	0.948	0.0051	−0.0697	3.8296	13.086	17.799	0.0533 ± 0.051	0.0003 ± 0.104	0.2152 ± 0.031	−0.999 ± failed	17,282.534	17,292.534
ADG1	model1	140.02				1984.1	2124.1	0.0659 ± 0.026				38,541.64	38,545.64
	model2	119.27			405.16	1591.5	2116	0.0564 ± 0.025		0.1915 ± 0.032		38,501.8	38,507.79
	model3	139.99	0.0035			1984.1	2124.1	0.066 ± 0.045	0.000 ± 0.013			38,541.64	38,547.64
	model4	152.79	0.2921	−5.6014		1978.5	2126	0.0719 ± 0.062	0.0001 ± 0.047		−0.838 ± failed	38,541.61	38,549.61
	model5	119.19	0.0013		405.06	1591.7	2115.9	0.0563 ± 0.040	0.000 ± 0.012	0.1914 ± 0.032		38,501.79	38,509.79
	model6	120.23	0.001	0.3467	405.55	1593.2	2119.3	0.0567 ± 0.049	0.000 ± 0.048	0.1914 ± 0.032	−1.000 ± failed	38,501.81	38,511.81
KR1	model1	0.1504				1.7	1.8505	0.0813 ± 0.028				7246.636	7250.636
	model2	0.1336			0.2721	1.4404	1.8461	0.0724 ± 0.027		0.1474 ± 0.033		7225.854	7231.854
	model3	0.1327	0.0074			1.7066	1.8467	0.0719 ± 0.045	0.004 ± 0.014			7246.53	7252.53
	model4	0.1252	0.0031	0.0063		1.7103	1.845	0.0679 ± 0.055	0.0017 ± 0.035		0.321 ± failed	7246.52	7254.52
	model5	0.1313	0.0010		0.2718	1.4415	1.8457	0.0712 ± 0.045	0.0005 ± 0.014	0.1473 ± 0.033		7225.856	7233.856
	model6	0.1304	0.001	0.0005	0.2717	1.442	1.8456	0.0707 ± 0.057	0.001 ± 0.037	0.1473 ± 0.034	0.041 ± failed	7225.856	7235.856
6MW	model1	0.4701				48.319	48.79	0.010 ± 0.033				4894.378	4898.378
	model2	0.3762			6.1416	42.243	48.761	0.008 ± 0.031		0.126 ± 0.118		4893.094	4899.094
	model3	0.4741	0.001			48.317	48.792	0.010 ± 0.035	0.000 ± 0.038			4894.378	4900.378
	model4	0.5019	0.001	0.0223		48.385	48.91	0.0103 ± 0.036	0.000 ± 0.181		0.9978 ± failed	4894.39	4902.39
	model5	0.3763	0.001		6.1428	42.243	48.763	0.008 ± 0.032	0.000 ± 0.040	0.126 ± 0.123		4893.096	4901.096
	model6	0.4103	0.001	0.019	6.2784	42.24	48.949	0.0084 ± 0.033	0.000 ± 0.178	0.1283 ± 0.124	0.940 ± failed	4893.12	4903.12
ADG2	model1	478.39				3415.7	3894.1	0.123 ± 0.092				9222.832	9226.832
	model2	496.09			11.855	3417.3	3925.2	0.1264 ± 0.095		0.003 ± 0.140		9222.898	9228.898
	model3	270.79	127.68			3453.2	3851.7	0.0703 ± 0.078	0.0331 ± 0.042			9222.266	9228.266
	model4	304.62	523.96	−399.34		3428.7	3857.9	0.079 ± 0.089	0.1358 ± 0.207		−1.000 ± failed	9221.762	9229.762
	model5	270.87	128.82		24.683	3472.6	3897	0.0695 ± 0.078	0.0331 ± 0.043	0.0063 ± 0.142		9222.428	9230.428
	model6	304.73	523.58	−399.4	0.0179	3428.6	3857.5	0.079 ± 0.089	0.1357 ± 0.207	0.000 ± 0.139	−1.000 ± failed	9221.762	9231.762
ADG3	model1	91.285				1483.4	1574.7	0.058 ± 0.067				8338.078	8342.078
	model2	77.436			174.94	1319.4	1571.8	0.0493 ± 0.062		0.1113 ± 0.122		8337.174	8343.174
	model3	69.365	20.03			1481.1	1570.5	0.0442 ± 0.065	0.0128 ± 0.039			8337.974	8343.974
	model4	61.819	8.2343	20.938		1480	1571	0.0394 ± 0.064	0.0052 ± 0.200		0.928 ± failed	8337.838	8345.838
	model5	75.273	2.2468		172.57	1321.3	1571.4	0.048 ± 0.069	0.0014 ± 0.040	0.110 ± 0.126		8337.174	8345.174
	model6	63.827	2.2126	11.862	162.08	1329.2	1569.2	0.041 ± 0.065	0.0014 ± 0.192	0.1033 ± 0.126	0.998 ± failed	8337.108	8347.108
KR2	model1	1.2795				5.7266	7.0062	0.1826 ± 0.103				2925.038	2929.038
	model2	1.4163			0.0026	5.9146	7.3355	0.1931 ± 0.108		0.0006 ± 0.142		2925.964	2931.964
	model3	0.6241	0.2951			5.9449	6.8641	0.0909 ± 0.096	0.043 ± 0.044			2924.254	2930.254
	model4	0.6061	0.9196	−0.5659		5.9033	6.8631	0.0883 ± 0.100	0.134 ± 0.178		−0.758 ± failed	2923.618	2931.618
	model5	0.624	0.2953		0.001	5.9462	6.8665	0.0909 ± 0.096	0.043 ± 0.045	0.0001 ± 0.142		2924.256	2932.256
	model6	0.6052	0.9215	−0.5678	0.001	5.9047	6.8647	0.0882 ± 0.100	0.1342 ± 0.178	0.0001 ± 0.141	−0.760 ± failed	2923.62	2933.62
KR3	model1	0.1064				0.4731	0.5796	0.1837 ± 0.096				447.652	451.652
	model2	0.0936			0.1303	0.3533	0.5773	0.1621 ± 0.095		0.2258 ± 0.121		444.804	450.804
	model3	0.041	0.0452			0.4801	0.5662	0.0724 ± 0.075	0.0798 ± 0.043			444.032	450.032
	model4	0.0443	0.0671	−0.0213		0.4783	0.5684	0.0779 ± 0.090	0.118 ± 0.144		−0.390 ± failed	443.406	451.406
	model5	0.043	0.0387		0.1021	0.3831	0.5669	0.0758 ± 0.077	0.0682 ± 0.044	0.1801 ± 0.125		442.308	450.308
	model6	0.0409	0.0568	−0.0166	0.0998	0.3855	0.5664	0.0722 ± 0.085	0.1003 ± 0.143	0.1762 ± 0.126	−0.346 ± failed	441.768	451.768

Abbreviations. BW: birth weight; WW: weaning weight; ADG1: average daily gain weight from birth to weaning; KR1: Kleiber ratio from birth to weaning (ADG1/WW0.75); 6MW: body weight at 6 months of age; ADG2: average daily gain weight from weaning to 6 months; ADG3: average daily gain weight from birth to 6 months; KR2: Kleiber ratio from weaning to 6 months; KR3: Kleiber ratio from birth to 6 months. $\sigma_{a}^{2}$: direct additive genetic variance; $\sigma_{m}^{2}$: maternal additive genetic variance; $\sigma_{c}^{2}$: maternal permanent environmental variance; $\sigma_{am}$: direct-maternal genetic covariance; $\sigma_{e}^{2}$: residual variance; $\sigma_{p}^{2}$: phenotypic variance; $h_{d}^{2}$: direct heritability; $h_{m}^{2}$: maternal heritability; $c^{2}$: ratio of maternal permanent environmental effect; $r_{am}$: direct-maternal genetic correlation; total heritability; S.E.: standard error; −2log⁡L: likelihood ratio test; *AIC*: Akaike information criterion.

**Table 5:** Likelihood ratio test results of early growth traits.

Model	DF	BW	WW	ADG1	KR1	6MW	ADG2	ADG3	KR2	KR3
1:2	1	116.472 ***	51.058 ***	39.846 ***	20.782 ***	1.284 ns	−0.066 ns	−0.002 ns	−0.926 ns	2.848 ns
1:3	1	15.464 ***	−0.004 ns	0 ns	0.106 ns	0 ns	0.566 ns	1.028 ns	0.784 ns	3.62 ns
1:4	2	21.040 ***	0.004 ns	0.022 ns	0.116 ns	−0.012 ns	1.07 ns	1.264 ns	1.42 ns	4.246 ns
1:5	2	133.948 ***	51.050 ***	39.846 ***	20.78 ***	1.282 ns	0.404 ns	1.026 ns	0.782 ns	5.344 ns
1:6	3	139.108 ***	51.246 ***	39.826 ***	20.78 ***	1.258 ns	1.07 ns	1.264 ns	1.418 ns	5.884 ns
2:5	1	17.476 ***	−0.008 ns	0 ns	−0.002 ns	−0.002 ns	0.47 ns	1.028 ns	1.708 ns	2.496 ns
2:6	2	22.636 ***	0.188 ns	−0.02 ns	−0.002 ns	−0.026 ns	1.136 ns	1.266 ns	2.344 ns	3.036 ns
3:4	1	5.576 *	0.008 ns	0.022 ns	0.01 ns	−0.012 ns	0.504 ns	0.236 ns	0.636 ns	0.626 ns
3:5	1	118.484 ***	51.054 ***	39.846 ***	20.674 ***	1.282 ns	−0.162 ns	−0.002 ns	−0.002 ns	1.724 ns
3:6	2	123.644 ***	51.250 ***	39.826 ***	20.674 ***	1.258 ns	0.504 ns	0.236 ns	0.634 ns	2.264 ns
4:6	1	118.068 ***	51.242 ***	39.804 ***	20.664 ***	1.270 ns	0 ns	0 ns	−0.002 ns	1.638 ns
5:6	1	5.160 *	0.196 ns	−0.02 ns	0 ns	−0.024 ns	0.666 ns	0.238 ns	0.636 ns	0.540 ns

Note: The means with different letters in each sub-class within a column differ significantly from another. ns: non-significant (*p* > 0.05).* *p* < 0.05, and *** *p* < 0.001.

**Table 6:** Heritability, genetic, and phenotypic correlation between early growth traits in Dumeng.

Trait	BW	WW	ADG1	KR1	6MW	ADG2	KR2	ADG3	KR3
BW	0.0352	−0.8748	−0.9548	−0.8029	−0.8244	0.2756	0.7300	−0.9787	−0.9909
WW	0.1890	0.0446	0.9859	0.9911	−0.2131	−0.9980	−0.9989	0.9953	0.9951
ADG1	−0.0302	0.9752	0.0564	0.9964	0.7623	−0.7568	−0.8522	0.8119	0.9645
KR1	−0.3665	0.8262	0.9213	0.0724	0.9185	−0.5000	−0.7088	0.9574	0.9646
6MW	0.0359	0.8397	0.8039	0.5936	0.0100	0.9978	0.9929	0.9998	0.9998
ADG2	0.0085	0.0582	0.0404	0.0351	0.5786	0.1230	0.9840	−0.1504	−0.1460
KR2	0.0031	−0.2927	−0.2988	−0.227	0.2482	0.9244	0.1826	−0.3544	−0.4427
ADG3	−0.1490	0.7836	0.8512	0.7439	0.9691	0.5544	0.2300	0.0580	0.9950
KR3	−0.5829	0.5767	0.8012	0.9033	0.7721	0.4477	0.1878	0.9023	0.1837

Note: The genetic correlation is above the diagonal, and the phenotypic correlation is below the diagonal.

## Data Availability

The data presented in this study are available from the corresponding authors upon reasonable request. The data are not publicly available due to privacy or ethical restrictions.
